# Hydrothermal Synthesis of Molybdenum Disulfide Quantum Dots for Highly Sensitive Detection of Iron Ions in Protein Succinate Oral Solution

**DOI:** 10.3390/mi14071368

**Published:** 2023-07-04

**Authors:** Yan Lang, Shuru Xu, Chunbin Zhang

**Affiliations:** 1 Department of Rehabilitation Therapy, Wuyi University, Nanping 354301, China; 2 Department of Medical Technology, Zhangzhou Health Vocational College/Collaborative Innovation Center for Translation Medical Testing and Application Technology, Zhangzhou 363000, China

**Keywords:** MoS_2_ quantum dots, fluorescent probe, Fe^3+^, performance, protein succinic acid oral solution

## Abstract

In this paper, a molybdenum disulfide fluorescent probe with an Fe^3+^ fluorescent system was first synthesized by the hydrothermal method for the detection of iron ion concentration in oral solution of protein succinate. It was characterized by infrared, fluorescence, X-ray photoelectron spectroscopy, scanning electron microscopy, and transmission electron microscopy. The probes were found to have good stability, photobleaching, and storage stability. The effects of dilution, pH, reaction time, and iron ion concentration on the fluorescent system were also investigated. The relative fluorescence intensity [(I_0_ − I)/I_0_] showed a good linear relationship with the iron ion concentration in the range of 0–50 μM, with the linear equation [(I_0_ − I)/I_0_] = 0.0148[Fe^3+^] + 0.0833 (r^2^ = 0.9943, n = 11) and the detection limit of 2.43 μM. The reaction mechanism was also explored, as well as its ion selectivity, reversibility, accuracy, precision, and concentration of Fe ions in the actual sample. It was found that the probe can selectively detect Fe ions with a certain degree of reversibility, accuracy, precision, and ideal recovery, and it can be used for the determination of Fe^3+^ in proteosuccinic acid oral solution.

## 1. Introduction

Metal ions are commonly present and play an important role in biology [1,2]. Iron is an essential element for human physiological activities and the most abundant transition metal element in living systems [3,4,5], playing a variety of physiological and pathological processes such as respiration, oxygen transport, enzymatic reactions, and energy production in combination with a critical oxygen role in various physiological and pathological processes such as respiration, oxygen transport, enzymatic reactions, and energy production by combining with oxygen [6,7,8,9]. Iron is one of the critical factors involved in the Fenton reaction [10], and the response of iron with hydrogen peroxide to generate reactive oxygen species (ROS) [11] causes cell damage and organ dysfunction [12,13]. For example, iron deficiency [14], caused when the iron level in the body is low, can lead to a decrease in the level of red blood cells or hemoglobin [15], i.e., iron deficiency anemia. Conversely, high iron levels in the body are considered to be “iron overload”. They can lead to liver disease [16], heart disease [17], diabetes [18], osteoarthritis [19], osteoporosis [20], metabolic syndrome [21], and other diseases, namely hemochromatosis [22]. Therefore, iron levels must be strictly regulated in human metabolism. Otherwise, abnormal conditions of iron concentration may lead to serious side effects. Thus, establishing a method to monitor Fe^3+^ levels in organisms is essential to assess the impact of iron on human health and disease states.

Currently, technical means have been developed for the analytical detection of Fe^3+^, mainly fluorescent sensors, atomic absorption (AAS), inductively coupled plasma mass spectrometry (ICP-MS), potentiometric solvation analysis (PSA), X-ray fluorescence spectrometry (XRF), and radioisotope labeling [23,24,25]. Among them, the fluorescence probe detection technique has recently become one of the most important means to detect the Fe^3+^ content in organisms or the environment because of its advantages such as high selectivity, simple operation, high sensitivity, visualization, and non-damage to the detection sample [26,27,28,29].

Disulfides (TMDs) have a large specific surface area and excellent semiconductor electronic properties, which have led to their widespread use [30,31,32,33]. Different structures of molybdenum disulfide exhibit other properties. Usually bulk or nanoparticle-like molybdenum disulfide is a semiconductor material with an indirect band gap of 1.29 eV and only exciton absorption does not have photoluminescent properties, when it has a plasmon resonance effect and can be applied to surface-enhanced fluorescence, photoelectric signal enhancement [34,35,36,37] and surface-enhanced Raman scattering [38].

When 2-D TMDs are compressed to the zero dimension, their quantum confinement and edge effects create an exceptional electronic and photophysical property [39]. In particular, MoS_2_ QDs are considered a promising compound due to their high stability, low toxicity, and excellent optical properties. Compared to other nanostructured MoS_2_, MoS_2_ QDs have large edge-to-volume ratios and high in-plane electron transport rates. MoS_2_ QDs have attracted much attention for their remarkable size-dependent optical and electronic properties, which have caused a great stir in many fields, including bioimaging [40,41,42], optical sensors [43,44,45,46], optoelectronic devices [47], photocatalysis [48,49,50], supercapacitors [51], and other fields.

l-cysteine (Cys), a common amino acid in living organisms, is the only α-amino acid containing reduced sulfhydryl groups among the 20 essential amino acids that make up the proteins of living organisms and is a non-essential amino acid [52]. Cys is very unstable and easily oxidized, whereas its hydrochloride form is relatively stable and can be stored for a long time [53]. Cys is a very important amino acid and is widely used in food processing [54], biomedicine [55], biochemical research [56], cosmetics [57], and other fields.

This paper prepares a molybdenum disulfide probe to determine iron ions in proteosuccinic acid oral solution, which will provide strong technical support for establishing a reliable, rapid, and low-cost method for analyzing middle iron elements.

## 2. Materials and Methods

### 2.1. Reagents and Instruments

Sodium molybdate, l-cysteine, Chlorobenzene, Potassium chloride, Sodium fluoride, Calcium chloride, Magnesium nitrate, Zinc sulfate, Ammonium chloride, Sodium bromide, Sodium carbonate, Sodium phosphate, Sodium sulfite, Cobalt Sulfate, Cadmium nitrate, Lead nitrate. The above reagents were purchased from Aladdin Reagent Network. All the above reagents are analytically pure and do not require further purification. Shanghai, China. Protein iron succinate oral solution was purchased from Jecheon Pharmaceutical Group Co. (Yunnan, China).

The following equipment was used: Phenom ProX Scanning Electron Microscope and Energy Spectrometer (Feiner Instruments, Eindhoven, The Netherlands), JEM-200CX transmission electron microscope (Rigaku Corporation, Beijing, China), F2500 Fluorescence Spectrophotometer (Hitachi, Ltd., Beijing, China), Thermo Scientific X-ray photoelectron spectrometer (Thermo Fisher, Louisville, KY, USA), Persee T9 UV-Visible Spectrophotometer (Shanghai Huyue Ming Scientific Instruments Co., Ltd., Shanghai, China), HJ-6A Combined Six Magnetic Stirrer (Changzhou Zhongbei Instrument Co., Ltd., Changzhou, China), and HH-1 Single-well Digital Display Thermostat Water Bath (Gongyi Honghua Instruments & Equipment Industry & Trade Co., Ltd., Gongyi, China).

### 2.2. Synthesis of Molybdenum Disulfide Probes

In this experiment, it was proposed to prepare MoS_2_ quantum dots by hydrothermal synthesis of Na_2_MoO_4_·2H_2_O and combine it with l-cysteine. A total of 0.3 g Na_2_MoO_4_·2H_2_O was dissolved in 25 mL of water, and the pH of the above solution was adjusted to 6.5 by ultrasonic shaking for 5 min. Then, 0.3 g of l-cysteine and 50 mL of water were added continuously to the previous solution, stirred, subjected to ultrasonic vibration for 10 min, and transferred to a 100 mL reaction kettle. The reaction kettle was placed in a furnace at 200 °C for 36 h. The solution was naturally cooled down and the supernatant containing MoS_2_ quantum dots was centrifuged at 9000 rpm for 10 min. The supernatant was cooled to room temperature, then filtered through a 0.22 μM disposable needle filter. The filtrate was dialyzed in a dialysis bag to obtain the desired molybdenum disulfide probe solution, which was stored in a refrigerator at 4 °C for the following experiments.

### 2.3. Characterization of Molybdenum Disulfide Probes

#### 2.3.1. Infrared, UV, and Fluorescence Spectroscopy

A small amount of sample and potassium bromide with a spectrum (wavelength 400–4000 cm^−1^) were ground uniformly, then compressed and tested on an infrared spectrophotometer (wavelength 400–4000 cm^−1^).

In the two cuvettes, the probe solution and an equal volume of ultrapure water of the metal to be measured were added to adjust the baseline, and then in the cuvette to be measured, a diluted Fe^3+^ solution with a concentration of 1 × 10^−4^ mol/L and the probe solution were added, and after the reaction of the system was complete, UV measurements were performed in the range of 200–700 nm.

The solutions of the probe and the metal to be measured were added to the colorimetric tube in order according to the desired ratio, and the fluorescence detection was performed after 10 min. The excitation wavelength of 300–500 nm, the emission wavelength of 300–600 nm, the sensitivity of 2, and the slit width of 10 nm were set for the fluorescence intensity measurement.

#### 2.3.2. X-ray Photoelectron Spectroscopy

Synthetic samples were tested by X-ray photoelectron spectroscopy using an aluminum target (Al Kα) as the excitation ray to analyze the elemental composition and chemical state of the sample.

#### 2.3.3. Scanning Electron Microscopy and Transmission Electron Microscopy

The specimens were glued to the specimen table and then put into the SEM at 15 KV for inspection. The synthetic material was tested by transmission electron microscopy with JEM-200CX, where the accelerating voltage was 200 kV.

### 2.4. Stability Studies

To verify the stability of the probe, we added it to different concentrations of NaCl solution and measured its fluorescence spectrum. Its fluorescence spectrum was measured under continuous light to examine the investigation’s photostability. Furthermore, the fluorescence spectra were measured under storage at 4 °C for one year.

### 2.5. Condition Optimization

To investigate the optimal experimental conditions of the probe for ferric ions, we diluted the probe solution to different multiples and measured its fluorescence spectrum. The effect of varying different pH environments on the probe solution was investigated in the range of 2.0~10.0. Meanwhile, the fluorescence spectra of the mixed solutions were measured at different reaction times after adding iron ions to the probe solution.

### 2.6. Basic Steps of Fluorescence Detection of Iron Ions

In this part of the experiment, a series of different concentrations of ferric ions were added to the diluted detection solution with 1 mL of 0.01 M (pH 7.0) NaAc-HAc buffer. Ultrapure water was added to a final volume of 5 mL, and the fluorescence intensity at 455 nm was measured at 370 nm after incubation for 15 min at room temperature.

### 2.7. Methodological Examination

In this part of the experiment, to test the probe’s precision, 10 sets of the same concentration of the probe mixed with iron ion solution were measured precisely and 1 mL of 0.01 M NaAc-HAc buffer (pH 7.0) was added. Ultrapure water was added to a final volume of 5 m. The fluorescence intensity at 455 nm was measured at an excitation wavelength of 370 nm and the relative standard deviation was calculated after incubation at room temperature for 15 min. To test the accuracy of the probe, three groups of fluorescent system solutions with iron ion solution concentrations of 3, 5, and 15 μM were taken, and the fluorescence intensity of each group was measured three times.

### 2.8. Detection of Iron Ions in Actual Samples

The following method was employed to analyze the ferric ion content in the oral solution of succinate proteins. A total of 2 mL of the oral solution of iron proteosuccinate was precisely pipetted into a 50 mL measuring flask, 2.0 mL of hydrochloric acid was added and well shaken. Then, water was added to the scale and well shaken. This was left for 30 min to obtain the test solution, which was diluted and prepared for use. The probe-containing answer solution next received 1 mL of the diluted sample, added after 15 min of incubation at room temperature. At this point, the fluorescence intensity at 455 nm was measured at the 370 nm excitation wavelength.

## 3. Results and Discussion

### 3.1. Characterization of Molybdenum Disulfide Fluorescent Probes

#### 3.1.1. Infrared Spectroscopy and Fluorescence Mapping

**Figure 1:**
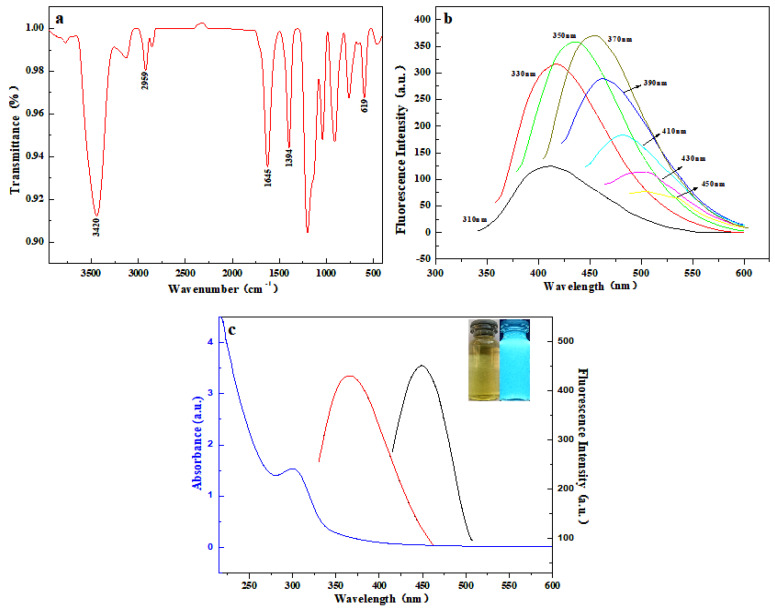
(**a**) Infrared spectrum of molybdenum disulfide probe. (**b**) Emission spectrum of molybdenum disulfide probe at different excitation wavelengths. (**c**) UV, excitation, and emission spectra of molybdenum disulfide probe (From left to right, UV, excitation, emission spectra).

The surface of the molybdenum disulfide quantum dots was characterized by FTIR spectroscopy. As seen in Figure 1a, the O-H vibrational peak and N-H stretching vibrational peak at 3420 cm^−1^ [58], the C-H vibrational peak at 2959 cm^−1^ [59], the asymmetric stretching vibrational peak C-NH at 1645 cm^−1^, 1349 cm^−1^, and the vibrational peak at 619 cm^−1^ are characteristic absorption peaks of Mo-S [60]. This suggests that its surface may be rich in functional groups such as carboxyl, amine, and hydroxyl. This is further evidence of the successful synthesis of molybdenum disulfide probes.

Figure 1b shows the emission spectra obtained by irradiating the probe solution with the light of different excitation wavelengths, from which it can be seen that the fluorescence intensity emitted tends to increase significantly. The most incredible fluorescence intensity is achieved at the excitation wavelength of 370 nm, which is when the excitation wavelength is gradually increased from 310 nm to 370 nm. However, the fluorescence intensity progressively drops when the excitation wavelength is gradually adjusted from 370 nm to 450 nm. The peak emission red-shift phenomenon of the emission spectrum when the excitation wavelength is changed from 310 nm to 450 nm. This reveals that the molybdenum disulfide quantum dots have an excitation wavelength dependence, which may come from the selective excitation of photons and also from the different size distribution of quantum dots.

One of the main features of MoS_2_ quantum dots is their good optical properties. At 365 nm in the UV (5 W), the described synthetic molybdenum disulfide probe shows a blue light and shows a yellowish color in the visible light (inserted in Figure 1c). As shown in Figure 1c, the fluorescence spectrum of the molybdenum disulfide probe has a maximum emission wavelength of 455 nm at an excitation wavelength of 370 nm.

#### 3.1.2. X-ray Photoelectron Spectroscopy

**Figure 2:**
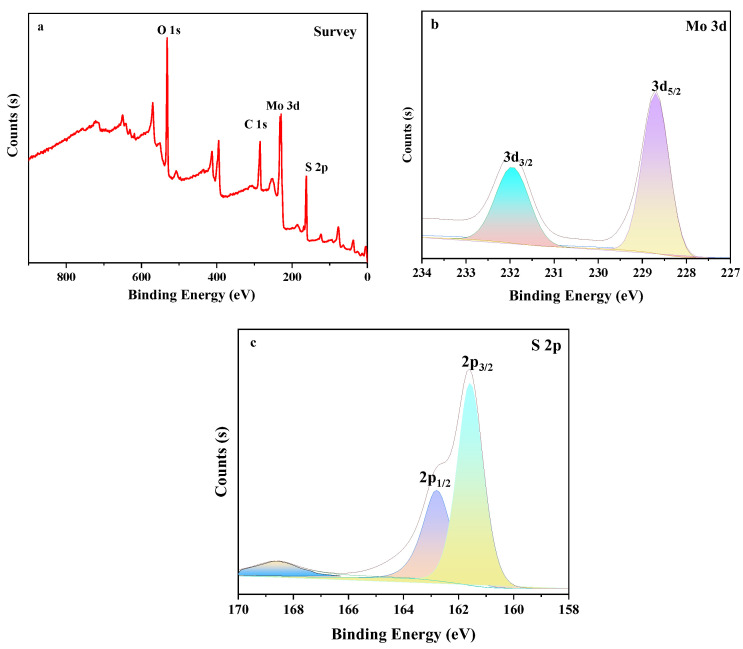
(**a**) Total XPS Spectrum of Molybdenum Disulfide Probe. (**b**) Peak Splitting of Mo 3d in Molybdenum Disulfide Probe. (**c**) Peak Splitting of S 2p in Molybdenum Disulfide Probe.

XPS measurements were used to study the molybdenum disulfide probe’s surface chemical composition and valence. As shown in Figure 2a, the probe is composed mainly of O, S, Mo, and other elements as seen in the XPS measurement spectra. The peaks appear at 161.43, 228.48 eV and are attributed to S 2p, Mo 3d, respectively. The spectra show peaks at 228.48 and 231.87 eV corresponding to Mo 3d_5/2_ and Mo 3d_3/2_ [61]. This indicates the dominance of the 4+ oxidation state of Mo [62,63]. The S 2p peaks at 161.43 and 162.43 eV are considered to be the result of the S 2p_3/2_ and S 2p_1/2_ orbitals of the divalent sulfide [64,65]. The peak at 168.8 eV is considered to be the oxidation state of S and is most likely attributed to the S_2_O_3_^2−^ group, which may have been formed by oxidation due to localized high temperatures during the experiment [66]. The resolved spectra of Mo 3d and S 2p confirmed the synthesis of the molybdenum disulfide probe.

#### 3.1.3. SEM and TEM

**Figure 3:**
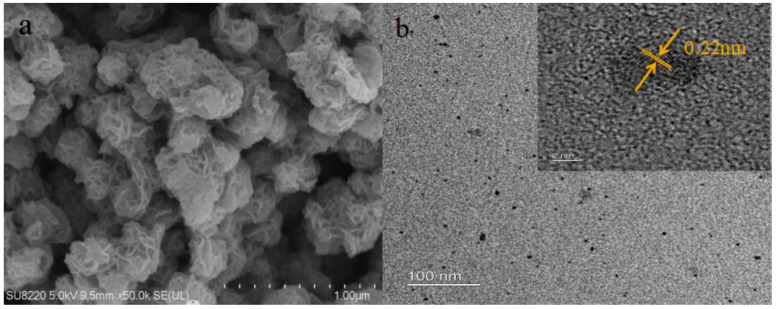
(**a**) SEM Spectra of Molybdenum Disulfide. (**b**) TEM Spectra of Molybdenum Disulfide.

SEM observed the morphology of the sample. Figure 3 shows the MoS_2_ prepared by the hydrothermal method. It can be seen that the MoS_2_ appears as homogeneous flakes which are aggregated together. Figure 3b shows the TEM pattern of MoS_2_, from which it can be seen that the prepared MoS_2_ is highly homogeneous and monodisperse. From the interpolation of the TEM image, it is clear that the lattice spacing of MoS_2_ is 0.22 nm, consistent with the (103) crystallographic plane reported in the literature [61]. Ultrathin molybdenum disulfide quantum dots with homogeneous particle size and narrow distribution were prepared.

### 3.2. Stability Study and Condition Optimization of Molybdenum Disulfide Fluorescent Probes

#### 3.2.1. Stability Studies

**Figure 4:**
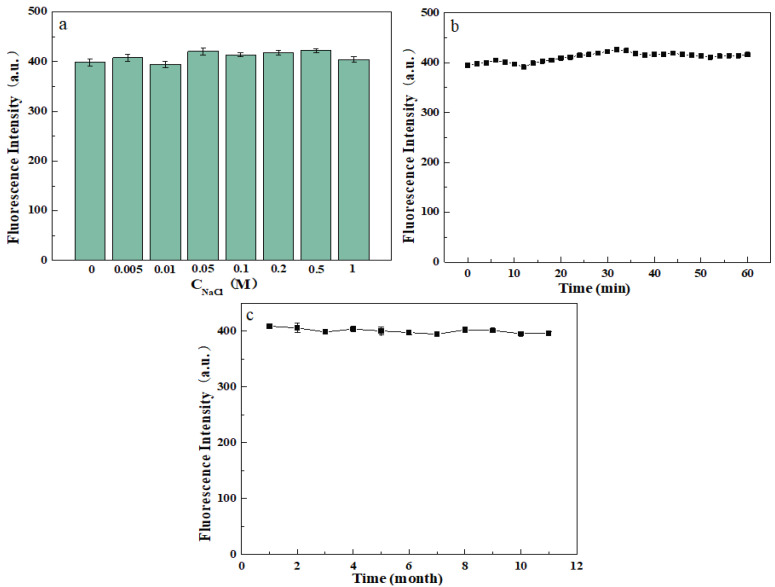
(**a**) Salt stability of the probe. (**b**) Photobleaching property of the probe. (**c**) Storage stability of the probe.

To verify the stability of the probe, we added it to different concentrations of NaCl solution and measured its fluorescence spectrum. As shown in Figure 4a, the probe maintained good stability when the concentration of NaCl solution was increased to 1 M. This indicates that the probe still has great potential for drug detection in a highly intense ionic environment. Meanwhile, the photostability of the probe was examined, and as shown in Figure 4b, the probe still maintained good stability under continuous irradiation for 60 min. In addition, the probe also has good storage stability, as shown in Figure 4c, and the fluorescence of the probe remained stable when stored at 4 °C for 1 year.

#### 3.2.2. Condition Optimization

**Figure 5:**
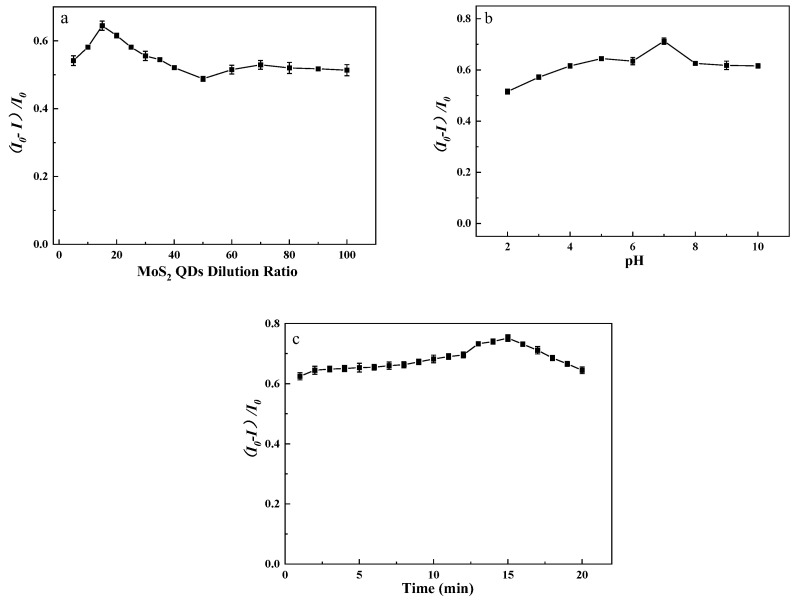
(**a**) Effect of different dilution ratios on fluorescence intensity (I_0_ and I, respectively, represent the fluorescence intensity of the probe solution before and after adding iron ions). (**b**) Effect of different pH on fluorescence intensity. (**c**) Effect of different reaction times on fluorescence intensity.

The fluorescence response of the molybdenum disulfide probe to iron ions was studied, and the fluorescence intensity of the probe changed significantly when appropriate concentrations of iron ions were added. On this basis, the experimental conditions such as the dilution times of the probe solution and the reaction time were optimized to explore the probe’s optimal experimental conditions for Fe ions. As shown in Figure 5a, the fluorescence intensity of the probe solution at different dilutions was measured. The results showed that the probe solution that diluted 15 times had the best testing effect. The effect of the solution pH was investigated in the range of 2.0~10.0 (2.0, 3.0, 4.0, 5.0, 6.0, 7.0, 8.0, 9.0, 10.0), as shown in Figure 5b, and the ideal pH level was determined to be 7.0. Meanwhile, the fluorescence intensity of the mixed solution was measured at different reaction times after adding iron ions to the probe solution, as shown in Figure 5c. The fluorescence intensity of the combined solution increased with reaction time, reaching its peak at 15 min. Therefore, the reaction time of this experiment was chosen at 15 min (I_0_ and I represent the fluorescence intensity of the probe solution in the absence and presence of iron ions, respectively).

#### 3.2.3. Basic Steps of Fluorescence Detection of Iron Ions

**Figure 6:**
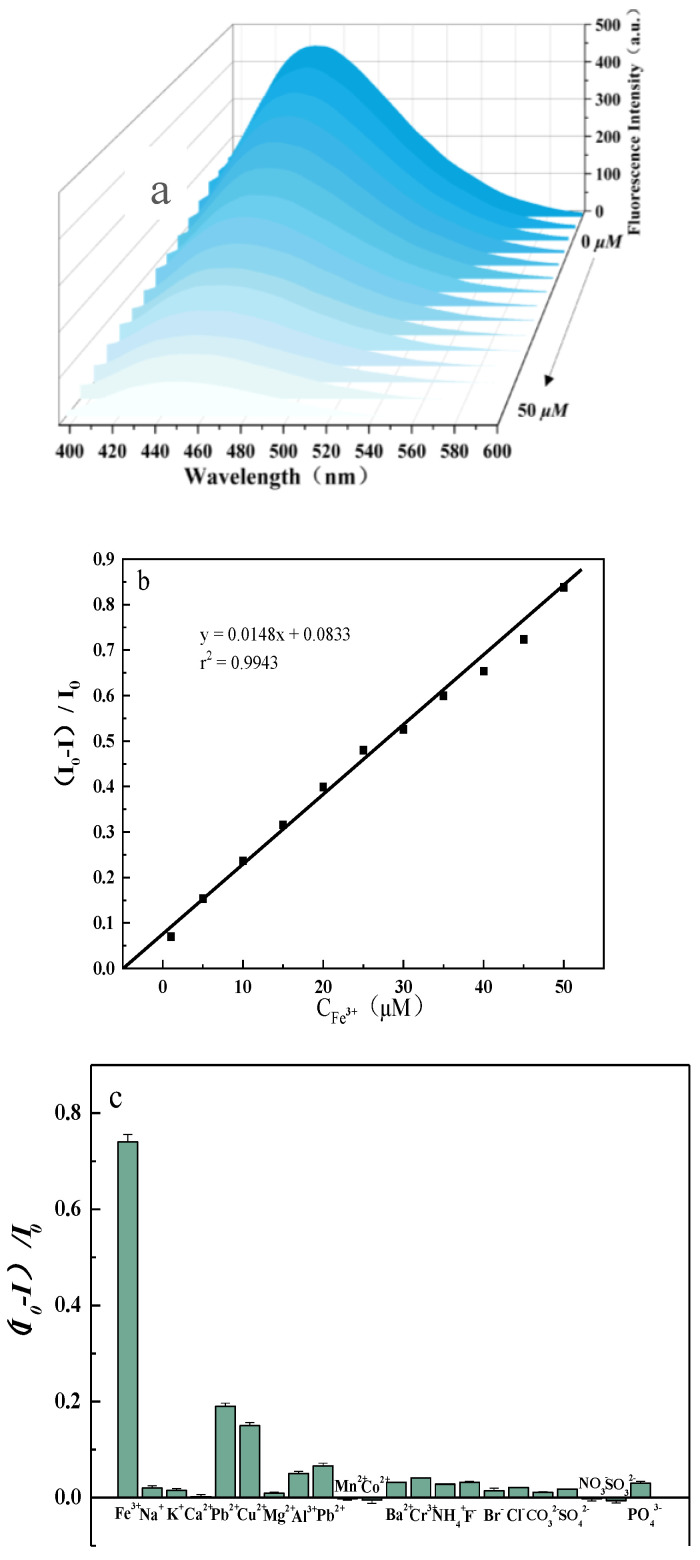
(**a**) Fluorescence Spectra of Different Concentrations of Iron Ions Added to the Probe Solution. (**b**) (I_0_ − I)/I_0_ Values and the Linear Relationship between the Concentration of Iron Ions. (**c**) (I_0_ − I)/I_0_ Values: Fluorescence Response to Different Ions.

As shown in Figure 6b, the fluorescence intensity of the probe decreased with the gradual increase in the concentration from 0 μM to 50 μM. Under the optimal experimental conditions, the relative fluorescence intensity [(I_0_ − I)/I_0_] showed a good linear relationship with the iron ion concentration in the range of 0–50 μM, with the linear equation [(I_0_ − I)/I_0_] = 0.0148[Fe^3+^] + 0.0833 (r^2^ = 0.9943, n = 11). The fluorescence intensity test of 1 μM probe solution was performed and the standard deviation (SD) was calculated based on its test results. The SD = 1.20% was calculated according to the standard deviation formula, and then the detection limit of the probe could be calculated as 2.43 μM from the formula of detection limit LOD = 3δ/k.

To evaluate the selectivity of the synthetic probes for detecting iron ions, we investigated the effect of other substances on the fluorescence intensity of the synthetic probes. These include Na^+^, K^+^, Ca^2+^, Mg^2+^, Al^3+^, Co^2+^, Pb^2+^, Mn^2+^, Ba^2+^, Cu^2+^, Cr^3+^, NH^4+^, F^−^, Br^−^, Cl^−^, CO_3_^2−^, SO_3_^2−^, SO_4_^2−^, NO_3_^−^, PO_4_^3−^, and some other common environmental ions. As shown in Figure 6c, 1 mM of some common ions and their analogs responded weakly to the probe but had almost no effect on the value of [(I_0_ − I)/I_0_]. In contrast, ferric ions responded significantly to the probe solution, and the results indicated that the probe could selectively detect ferric ions.

**Figure 7:**
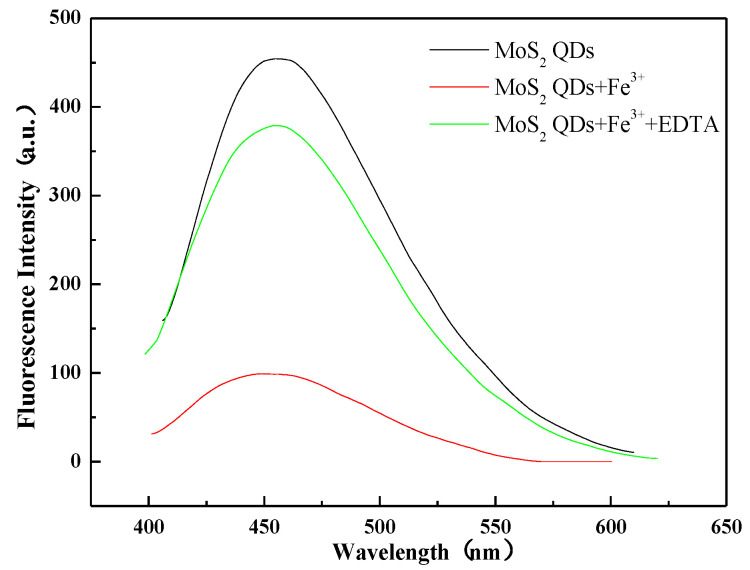
Fluorescence spectra of molybdenum disulfide probe with Fe^3+^ added to EDTA after reaction.

Figure 7 shows the fluorescence spectra of the molybdenum disulfide probe when EDTA was added after the reaction of the probe with Fe^3+^. In order to test whether the reaction of the molybdenum disulfide probe with Fe^3+^ is reversible, we designed an EDTA reversibility experiment. We conducted a fluorescence test after 5 min of the reaction of the molybdenum disulfide probe with Fe^3+^ and measured the fluorescence value after adding EDTA for 1 h. The fluorescence enhancement was found to be nearly 4-fold, indicating that the reaction of the molybdenum disulfide probe with Fe^3+^ has certain reversibility.

### 3.3. Exploration of Mechanism

Molybdenum disulfide consists of three atomic planar layers stacked on each other. It has a large specific surface area and high electron migration rate, so we speculate that in the mixed solution of iron ions and molybdenum disulfide probe, there is electron transfer between iron ions and the surface of molybdenum disulfide quantum dots, which leads to the fluorescence burst of molybdenum disulfide quantum dots. When EDTA solution is added, EDTA forms a complex with the iron ions on the surface of the molybdenum disulfide quantum dots, thus allowing the fluorescence of the molybdenum disulfide quantum dots to be restored.

### 3.4. Methodological Examination

#### 3.4.1. Precision Experiment

In order to test the precision of the probe, 8 groups of equal volume and concentration of the probe solution were configured. As shown in Table 1, and 1 mL of each was added to a solution with a concentration of 10 μM ferric ions, and the fluorescence intensity was tested after 15 min. I_0_ in the table is the fluorescence value when the probe is a blank solution, I is the fluorescence value of the solution after the probe is thoroughly combined with the ferric ion solution, and ∆I is the difference between I_0_ and I. The fluorescence intensity at 455 nm was recorded to calculate the relative standard deviation RSD = 0.47%, and the experimental data indicate that the probe measurement of iron ions can be used with a small error in the detection method.

#### 3.4.2. Accuracy Test

Table 2 shows the experimental results of the accuracy of the molybdenum disulfide probe for measuring ferric ions. Equal volumes of 0.5 × 10^−5^, 2.5 × 10^−5^, and 4.5 × 10^−5^ mol/L were selected to measure the content of 10 × 10^−6^ mol/L of iron ions, and the fluorescence intensity at 455 nm was recorded and the recoveries and relative standard deviations were calculated to test the accuracy of the composite probe for measuring iron ions. The experimental results are shown in Table 2, with the average recoveries of 94.2–95.5% and the calculated relative standard deviations of 1.52–1.84% were less than 2.0%. The experimental data indicate that the accuracy of the probe assay for measuring iron ions is high.

#### 3.4.3. Detection of Iron Ions in Actual Samples

To evaluate the utility of the synthetic probe, we applied the method to detect ferric ions in the solution of ferric proteins succinate oral solution samples. We used the spiked recovery method; the measurement results are shown in Table 3. The recoveries of the samples in the oral solution of iron proteosuccinate samples ranged from 91.3% to 98.1% with relative standard deviations of 0.41% to 0.63%, which were satisfactory.

## 4. Conclusions

This paper successfully synthesized molybdenum disulfide probes by a one-step hydrothermal method using Na_2_MoO_4_·2H_2_O as the molybdenum source and l-cysteine as the sulfur source. It can detect for the detection of iron ions in the oral solution of protein succinate. The probe was found to have good salt stability, photobleaching properties, and storage stability. The best results were obtained at a probe dilution of 15 times, pH 7, and a reaction time of 15 min. The relative fluorescence intensity [(I_0_ − I)/I_0_] showed a good linear relationship with the iron ion concentration in the range of 0–50 μM, with the linear equation [(I_0_ − I)/I_0_] = 0.0148[Fe^3+^] + 0.0833 (r^2^ = 0.9943, n = 11) and the detection limit of 2.43 μM. It also has a certain degree of reversibility, can selectively detect iron ions, accuracy, precision is good, for the actual sample in the recovery of 91.3%~98.1%, the relative standard deviation of 0.41%~0.63%, the recovery effect is ideal. The probe has the advantages of simple preparation, good selectivity, low detection limit, and wide linear range, which can effectively overcome external environmental factors’ interference with the detection results.

## Figures and Tables

**Table 1:** Precision test results of molybdenum disulfide fluorescence probe.

Number of Measurements	∆I (I_0_ − I)	∆I Average Value	RSD (%)
1	205.33		
2	208.45		
3	206.31		
4	206.84	206.34	0.47
5	205.76		
6	206.47		
7	205.91		
8	205.66		

**Table 2:** Accuracy test results of molybdenum disulfide fluorescence probe.

Probe Concentration (mol/L)	Recover Amount (mol/L)	Recovery Rate (%)	Average Recovery Rate (%)	RSD (%)
	9.66 × 10^−6^	96.6		
0.5 × 10^−5^	9.32 × 10^−6^	93.2	95.1	1.84
	9.56 × 10^−6^	95.6		
	9.24 × 10^−6^	92.4		
2.5 × 10^−5^	9.62 × 10^−6^	96.2	95.5	1.52
	9.38 × 10^−6^	93.8		
	9.54 × 10^−6^	95.4		
4.5 × 10^−5^	9.26 × 10^−6^	92.6	94.2	1.55
	9.47 × 10^−6^	94.7		

**Table 3:** Analysis results of actual samples.

Sample	Add Amount (mol/L)	Found (μM) (mol/L)	Average Recovery (%)	RSD (%, n = 3)
1	1 × 10^−6^	0.9 ± 0.2	95.8%	1.42
2	5 × 10^−6^	4.9 ± 0.2	95.6%	1.26
3	10 × 10^−6^	9.8 ± 0.2	94.6%	1.16
